# Readability of Ebola Information on Websites of Public Health Agencies, United States, United Kingdom, Canada, Australia, and Europe

**DOI:** 10.3201/eid2107.141829

**Published:** 2015-07

**Authors:** Enrique Castro-Sánchez, Elpiniki Spanoudakis, Alison H. Holmes

**Affiliations:** Imperial College London, London, UK; and National Institute for Health Research, London

**Keywords:** Ebola, Ebola virus, viruses, information, readability, public, health literacy, suitability, websites, United States, United Kingdom, Canada, Australia, Europe

## Abstract

Public involvement in efforts to control the current Ebola virus disease epidemic requires understandable information. We reviewed the readability of Ebola information from public health agencies in non–Ebola-affected areas. A substantial proportion of citizens would have difficulty understanding existing information, which would potentially hinder effective health-seeking behaviors.

The outbreak of Ebola virus disease (EVD) that originated in Guinea in April 2014 has become the largest known epidemic of this pathogen and was declared an international public health emergency (*1*). In addition, repatriation of health care workers and volunteers to Europe and the United States has resulted in human-to-human transmission in western health care organizations (*2*), thus bringing Ebola to the fore of public attention in settings far removed from local outbreak areas.

Currently, because there is no antiviral treatment or vaccine, surveillance and strict observation of recommended infection prevention and control measures, aided by public awareness regarding symptoms and prompt health care–seeking behavior, are essential efforts to control Ebola. In Africa, low awareness has led to community misunderstandings and unwillingness to cooperate with medical teams (*3*). In non–EVD-affected countries, nonrigorous information has resulted in unfounded fear among health care workers and citizens, disrupting the activity of hospitals caring for persons with EVD (*4*).

For health messages to be followed effectively, they must be tailored to the health literacy of the audience. Health literacy, which refers to “the cognitive and social skills which determine the motivation and ability of individuals to gain access to, understand and use information in ways which promote and maintain good health” (*5*), has been associated with better self-care (*6*). However, a substantial proportion of citizens worldwide have insufficient or inadequate health literacy (*7*).

Several factors, including readability of information provided (*8*), can help reduce health literacy deficits. Readability refers to “the determination of the reading comprehension level a person must have to understand written materials” (*9*). It is recommended that health information materials should be written at a level typically understandable by an 11-year-old person (*10*). Such recommendations for clarity and understandability might be more effective if one considers that anxiety or panic attributed to a highly virulent infection, such as Ebola, might hinder comprehension of related information (*11*).

We examined readability of EVD public information available from selected public health agencies in non–EVD-affected countries. Countries that have EVD should explore how well this information would serve to reduce panic and anxiety and perform as an effective source of advice for the public.

## The Study

Current information on Ebola aimed at the public was downloaded from various websites; a list is provided in Technical Appendix. Information was retrieved from the European Centre for Disease Control (Ebola factsheet for the general public); the US Centers for Disease Prevention and Control (CDC; Questions and answers on Ebola); Public Health England (PHE) in the United Kingdom (Ebola: public health questions and answers); and the government of Canada (Ebola virus disease) on September 1, 2014 and from the government of Australia (Ebolavirus disease outbreaks in West Africa: important information for travellers, patients and consumers) and the World Health Organization (WHO; Advice for individuals and families. Ebola guidance package) on November 11, 2014.

Any figures, such as maps or pictograms, were removed, and content was then formatted as plain text and uploaded to a free online tool (http://www.readabilityformulas.com/free-readability-formula-tests.php/) from which different readability indicators were obtained (Technical Appendix). The causes, symptoms, risks, treatment, prevention, and surveillance pages in the Canadian website were individually opened and analyzed. We calculated measures of central tendency and dispersion for scores obtained in indicators reported by using Stata version 10.1 (StataCorp LP, College Station, TX, USA).

Results are shown in the Table. In terms of reading difficulty, mean Flesch Reading Ease score for all information was 48.85 (SD 7.76; 95% CI 40.69–57.00) and indicated difficult to read. WHO information was easiest to read (score 62.3); information from Australia was most difficult to read (score 42). Mean Gunning FOG Index was 12.6 (SD 1.68; 95% CI 10.83–14.36) and indicated difficult to read. Again, written content from WHO and Australia were at the easiest and most difficult reading levels, respectively.

**Table:** Readability of Ebola public information published by selected public health agencies*

Readability formula	Selected website						Mean ± SD (95% CI)
	ECDC (20.0)†	PHE (16.40)†	CDC (17.49)†	Government of Canada (16.38)†	WHO (NA)	Government of Australia (12.55)†	
Gunning Fog Index	13.7 (hard to read)	13.9 (hard to read)	10.7 (hard to read)	12.9 (hard to read)	10.3 (fairly easy to read)	14.1 (hard to read)	12.6 ± 1.68 (10.83–14.36)
Flesch Reading Ease Score	48.2 (difficult to read)	45.4 (difficult to read)	53 (fairly difficult to read)	42.2 (difficult to read)	62.3 (standard/avg)	42 (difficult to read)	48.85 ± 7.76 (40.69–57.00)
Automated Readability Index	11.6 (17–18 y old)	12.5 (18–19 y old)	7.8 (12–14 y old)	11.8 (17–18 y old)	8.6 (13–15 y old)	11.9 (17–18 y old)	10.7 ± 1.97 (8.62–12.77)
Coleman-Liau Index	12 (12th grade)	12 (12th grade)	10 (10th grade)	13 (college)	9 (9th grade)	11 (11th grade)	11.16 ± 1.47 (9.62–12.71)
SMOG Index	10.7 (11th grade)	11 (11th grade)	9.4 (9th grade)	11.1 (11th grade)	8.4 (8th grade)	11.5 (12th grade)	10.35 ± 1.19 (9.09–11.60)
Linsear Write Formula	13 (college)	14.1 (college)	8.4 (8th grade)	12.6 (college)	9.5 (10th grade)	14.1 (college)	11.95 ± 2.42 (9.40–14.49)
Flesch-Kincaid US Grade Level	11.3 (11th grade)	12.1 (12th grade)	9.2 (9th grade)	11.8 (12th grade)	8.8 (9th grade)	12.4 (12th grade)	10.93 ± 1.54 (9.31–12.55)

*ECDC, European Centre for Disease Control; PHE, Public Health England; CDC, US Centers for Disease Control and Prevention; WHO, World Health Organization; NA, not applicable; avg, average; SMOG, simple measure of gobbledygook. Items in parentheses are general assessments, age levels, or US-equivalent grade levels. †Percentage of adults 16–65 years of age with literacy proficiency below reading level recommended for health information materials. ECDC percentage refers to a sample of 17 European Union Member States (*12*).

Factsheets from PHE and Canada required a 12th US school grade reading level to be understood, and the CDC and WHO factsheets required a 9th US school grade reading level. Comparable results were obtained with Coleman-Liau Index and the SMOG (simple measure of gobbledygook) formula. The Automated Readability Index for all materials was 10.7 (SD 1.97; 95% CI 8.62–12.77) and required an age of 15–16 years to understand the text. Finally, information from PHE and Australia was written at the most demanding level according to the Linsear Write Formula (score 14.1, or college level), and the CDC content required an 8th US grade reading level for comprehension (score 8.4). The mean result for all content was 11.95 (SD 2.42, 95% CI 9.40–14.49).

## Conclusions

Our analyses indicate that the information on EVD provided on websites of different public health agencies is written at a higher than recommended reading level. For such a reason, a substantial proportion of citizens with low literacy in the United States, United Kingdom, Canada, Australia, and Europe would have difficulty understanding key EVD messages. These results are of concern because poor readability might prevent or delay adoption of appropriate health-seeking behaviors, prolong ineffective self-care strategies, and perpetuate stigmatizing attitudes toward Ebola.

Providing adequate EVD information for the public might be arduous. Uncertainties remain regarding optimal clinical management for Ebola patients and disagreements in infection prevention and control protocols. The continued modification of procedures also demands constant public engagement efforts to avoid dissemination of conflicting messages and to ensure that information released is up to date and presented at a level that can be adequately understood. Because there have been limited national communication campaigns in non–EVD-affected countries, it is likely that other outlets, including traditional mass media and social media, might have been used by the public to meet their information needs (*13*), with probable trade-offs between immediacy and accuracy or reliability of information provided. The variation of readability identified in our study suggests that with contributions from health literacy specialists, public health agencies could further adapt the EVD information provided.

We recognize that persons accessing health information online are not representative of the average population because they are more educated and benefit from better information-seeking skills and health literacy (*14*). Thus, the online audience might be able to make more effective use of information on websites analyzed. However, such might not be the case for persons whose first language is not English, who might find information provided even more difficult to understand because of linguistic and cultural barriers.

It is accepted that readability measures alone may not reflect the level at which information is written (*15*). Because the Ebola epidemic has continued since our analysis, it might be possible for currently available information to have been modified and display greater readability. Our analysis was not exhaustive because we assessed selected public health agencies in non–EVD-affected countries and concentrated in English language materials. Therefore, our findings might not be representative of all health pages with EVD information. However, we evaluated key official websites.

Public health agencies in non–EVD-affected countries must improve the readability of EVD information currently provided so that the public could adopt effective self-care strategies, avoid fear, and reduce unnecessary panic and stigma toward persons affected by Ebola. In addition, agencies should consider multimodal Ebola awareness campaigns, including social marketing interventions, to encourage and strengthen public participation in Ebola control efforts.

**Technical Appendix.** Website addresses for information on Ebola and readability indicators.
