# Supplementary material for: Readability of Ebola Information on Websites of Public Health Agencies, United States, United Kingdom, Canada, Australia, and Europe
Source: Emerg Infect Dis. 2015 Jul;21(7):1217–9. doi: 10.3201/eid2107.141829 (PMC4480387; doi:10.3201/eid2107.141829)
Supplement: Supplementary file 1 — Technical Appendix. Website addresses for information on Ebola and readability indicators. [file 14-1829-Techapp-s1.pdf]

Article DOI: http://dx.doi.org/10.3201/eid2107.141829

Readability of Ebola Information on Websites of Public Health Agencies, United States, United Kingdom, Canada, Australia, and Europe

Technical Appendix

Websites Reviewed

European Centre for Disease Control. Ebola factsheet for the general public [cited 2014 Sep 1]. http://www.ecdc.europa.eu/en/healthtopics/ebola_marburg_fevers/factsheet_general_public/Pages/factsheet-general-public.aspx

Centers for Disease Prevention and Control. Questions and answers on Ebola [cited 2014 Sep 1]. http://www.cdc.gov/vhf/ebola/outbreaks/2014-west-africa/qa.html

Public Health England. Ebola: public health questions and answers [cited 2014 Sep 1]. https://www.gov.uk/government/uploads/system/uploads/attachment_data/file/370777/141104__Ebola_QA_For_Public_LF.pdf

Government of Canada. Ebola virus disease [cited 2014 Sep 1]. http://healthycanadians.gc.ca/diseases-conditions-maladies-affections/disease-maladie/ebola/index-eng.php

Government of Australia. Ebolavirus disease outbreaks in West Africa – important information for travellers, patients and consumers [cited 2014 Nov 11]. http://www.health.gov.au/internet/main/publishing.nsf/Content/2974D69E347C60F6CA257D1E00106707/$File/ebola-travellers-patients-consumers.pdf

World Health Organization. Advice for individuals and families. Ebola guidance package [cited 2014 Nov 11]. http://apps.who.int/iris/bitstream/10665/136474/1/WHO_EVD_Guidance_AdviceFam_14.1_eng.pdf?ua = 1

Readability Indicator Definitions

Gunning FOG Index, which correlates aspects of a text with its grade level.

Flesch Reading Ease score, which measures readability on a scale from 0 to 100 (100 being easiest to read, where a score of 60–70 is considered well written and easy to follow by the average reader).

Automated Readability Index, which is influenced by sentence length and character count, with shorter sentences and words lowering the score.

Coleman-Liau Index measures the understandability of a text and outputs the US grade level considered necessary to comprehend a text.

SMOG (simple measure of gobbledygook) formula estimates the years of education needed to fully understand a piece of writing.

Linsear Write Formula, which was developed to calculate the readability of technical manuals, with shorter sentences and less complex words lowering the score.

Flesch-Kincaid US Grade Level, which calculates a readability score by using a combination of word– and syllable–sentence proportions and outputs the number of years of US education required to understand a text.
